# Diagnosing allergic sensitizations in the third millennium: why clinicians should know allergen molecule structures

**DOI:** 10.1186/s13601-017-0158-7

**Published:** 2017-07-17

**Authors:** C. Alessandri, R. Ferrara, M. L. Bernardi, D. Zennaro, L. Tuppo, I. Giangrieco, M. Tamburrini, A. Mari, M. A. Ciardiello

**Affiliations:** 1 CAAM - Centri Associati di Allergologia Molecolare, Rome, Italy; 2 Istituto di Bioscienze e Biorisorse - IBBR-CNR, Naples, Italy; 3 Allergy Data Laboratories s.c., Latina, Italy

**Keywords:** Allergenic molecules, Allergenic extracts, Nanotechnology, Arrayed allergens, Multiplex diagnosis, Allergen epitope profile

## Abstract

Diagnostic tests to detect allergic sensitization were introduced at the end of the nineteenth century but only in the late 1990s did the advent of molecular allergology revolutionize the approach to the allergic patient. Personalized Medicine, a medical procedure that separates patients into different groups with different medical decisions, practices and interventions has sanctioned this change. In fact, in the last few years molecular allergology and the observation that not every patient has the same allergic profile, even when allergic to the same allergenic source, has originated the concept “one size does not fit all”. This new approach requires the identification of still unknown allergens, but also the more detailed investigation of those already known. In depth studies of the structure–function relationships in allergenic molecules can reveal the structural determinants involved in the IgE-binding. Then, the knowledge of the epitope profile of each allergen and of the environmental/experimental conditions affecting the exposure of IgE-binding epitopes can provide important contributions to the understanding of cross-reaction processes and to the improvement of diagnosis, immunotherapy and the overall patient treatment. The evolution of diagnostic systems cannot ignore these new needs in this field.

## Background

The last decades have seen a sharp increase in the prevalence of allergic diseases in all countries, affecting both children and adults [1, 2]. The World Health Organization considers allergy a non-transmittable disease which is out of control. Among the various existing forms of allergy, the most common are those determined by the production of IgE towards otherwise innocuous compounds, causing diseases like asthma, rhinitis, urticaria, anaphylaxis, eczema and conjunctivitis. Although sometimes and in selected cases specific immunotherapy can be prescribed towards inhalant and food allergens, when possible avoiding the allergy triggers or decreasing the exposure can contribute to reducing the symptoms. This implies that a correct allergy diagnosis is of crucial importance in the definition of the treatment plan for each allergic subject.

## What we know about allergy diagnosis

The skin test (ST) is historically the basic tool for allergy diagnosis, not developed further since its introduction in medicine at the end of the nineteenth century. ST, performed using allergenic extracts, has many limitations: it is an operator-dependent in vivo test, it is not riskless and it is limited to the use of some allergenic extracts [3] and excludes all allergenic molecules as they are not allowed to be applied to humans in vivo. However, the composition of the used extracts can be variable, mostly depending on the protocols used for the extraction, on proteolytic degradation and on the quality of the starting material. In the case of fruit extracts, it depends also on the ripening stage, post-harvest treatments and differences among cultivars [4–8]. Allergen extracts commercially available in the EU are standardized in terms of total allergenic activity by measuring the overall IgE-binding potency but not the specific allergenic protein content. This makes it impossible to compare and interchange extracts produced by different companies from the same allergenic source [9].

 An important issue linked to ST is the safety of the procedure. In fact, anaphylaxis after ST is a rare but possible event, especially in patients with a history of severe allergic reactions or even in asymptomatic pediatric patients [10] and some cases are described in literature induced by the allergenic source used in the prick–prick test [11–13].

A further concern about the ST is linked to the unknown function of certain molecules contained in the extracts that can interfere with the subject reactivity. For instance, histidine is a precursor of histamine and its content depends on the species [14]. It is easily released in the presence of bacterial histidine decarboxylase, and specific environmental conditions. In this case the ST could provide false positive results. Moreover, the reactivity of some allergenic proteins could vary if the ST is performed with raw or cooked food material [15, 16]. For all these reasons the detection of specific IgE in peripheral blood using allergenic proteins should be preferred [17, 18].

During the seventies IgE detection in blood was introduced. Since that time the laboratory allergy test has been greatly developed, leading to third generation tools. This implies, however, increasing testing costs, particularly in the case of patients needing IgE detection for several allergenic sources, each one to be individually performed (i.e. the singleplex test). The introduction of allergenic molecules in the allergy diagnosis enables the identification of genuine primary sensitization, otherwise impossible using allergen extracts. The use of allergenic molecules started in the late nineties and improved the quality of the lab testing compared to the traditional ST. However, the costs sharply increased leading to its use only for refining the allergy diagnosis and not as the natural basis of it. Furthermore, a large amount of blood is required to test a few extracts or allergenic molecules. To overcome the above limitations and make allergenic molecules available in routine diagnosis, decreasing at the same time the overall cost, microarray technology started to be used in lab allergy diagnosis (i.e. the multiplex test) more than ten years ago. Testing a small blood sample for IgE towards a combination of dozens of allergenic molecules arrayed on a biochip brought allergy diagnosis to the next level [19–21].

After ten years of rapid development resulting in the first generation multiplex allergy test (i.e. ISAC) increasing from 74 to 112 allergenic molecules, no further developments in its use for in routine diagnosis have been realized in the last five years.

## What has been shown

### Sensitization does not necessarily predict allergy

Allergens are proteins contained in allergenic sources; sensitization occurs when specific IgE are produced by atopic individuals and bind the trigger molecules. Allergy is an abnormal immunological reaction occurring in sensitized patients exposed to an allergen. Sensitization and allergy, although being very often correlated, are not always fully comparable: a positive test result (sensitization) is likely to correspond to a clinical reaction, but this cannot be considered valid in all cases [22–24].

### The best possible allergy diagnosis cannot be restricted to a few protein families

**Fig. 1:**
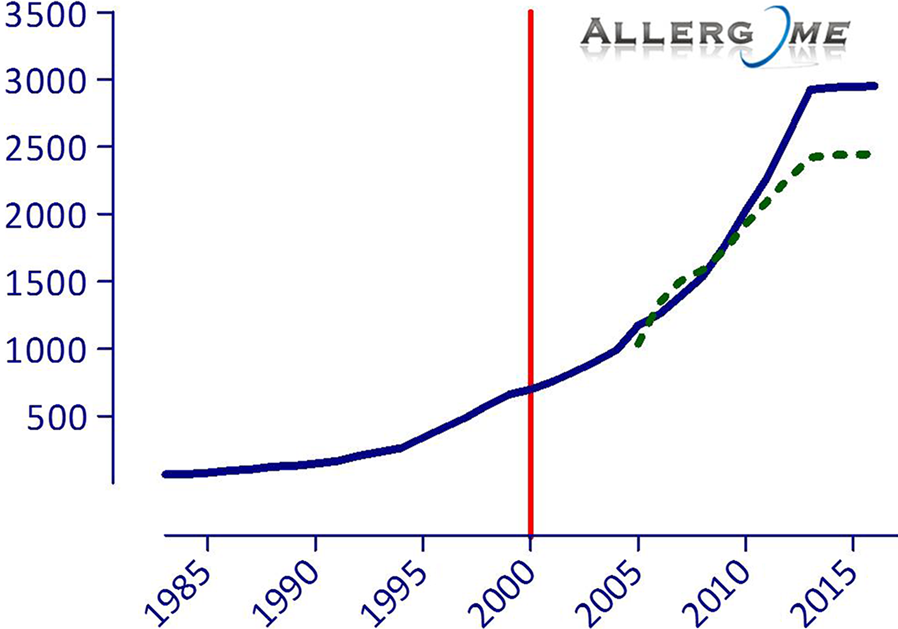
Scientific publications in molecular allergology in the last decades

**Fig. 2:**
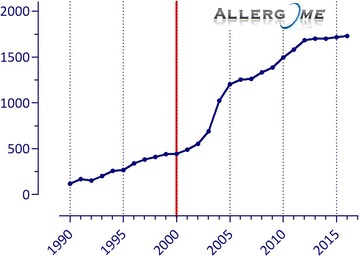
Time trend of allergenic sources (*dashed line*) and molecule identification (*straight line*) in the last decades

Years ago the majority of allergic reactions used to be attributed to the allergenic proteins included in a restricted number of protein families [25]. A limited spectrum of individual allergenic molecules, considered as representatives of the above families or those tightly belonging to a single allergenic source, were then selected and used for the so-called component resolved diagnosis (CRD). Nowadays, both the number of known allergenic proteins and the number of protein families they belong to are increasing (Figs. 1, 2). A comprehensive and updated overview is available from the Allergome platform [26].

Among the many new allergens identified, some of them belong to new families of allergenic proteins, such as Pun g 7 [5] and Pru p 7 [27–29], which are members of the gibberellin-regulated protein family [30], which is reported to display antimicrobial activity [31]. Quite recently the 7 kDa LTP sub-family has been included in the list of those recorded to be allergenic. In fact, in addition to the long list of 9 k-LTPs reported as allergenic proteins [32], two 7 k-LTPs, Api g 6 from the celery tuber [33] and Sola l 6 from tomato seeds [20] have been registered by the IUIS as allergens. Widening our view, we should add the major allergen of red meat, α-Gal, that is an oligosaccharide bound to some mammalian proteins, except those from some non-human primates and humans [34]. These are just some examples of allergenic proteins belonging to new allergenic families to which the human being is constantly exposed.

In summary, the available knowledge in this field seems to support the concept that “each protein is a potential allergen”. As a matter of fact, many proteins have so far been classified as “allergenic”, whereas none has been definitely classified as “non-allergenic”. It happens that some proteins, appearing not to be allergenic when a few subjects are tested, are recognized as allergens when a larger population, or a population selected on the basis of different criteria, is analyzed [35]. It is also possible that some factors could have biased the process of allergen identification directing researchers towards selected molecules. For instance, a high concentration in the natural source has made the isolation and characterization of some molecules easier. Moreover, the exploitation of recombinant DNA technology has prompted the identification and characterization of many homologs of already known allergens [36]. The high prevalence of IgE detection towards some specific allergens (major allergens), compared to others detected in the same source with only a minority of patients sensitized (minor allergens), can be an additional factor causing unintentional selections in the identification of allergenic proteins. For instance, Pru p 3 is the most abundant protein in peach peel and is also the one most frequently causing allergic reactions to peach [37]. This allergen has been known for a long time, whereas Pru p 7, which is less abundant in peach and shows an IgE detection prevalence lower than Pru p 3, has been identified very recently [27, 28]. It is worth noting that the late identification of an allergen does not mean that it does not cause severe reactions.

For all the above-mentioned reasons, we would suggest that “molecule based diagnosis (MBD)” definition, which does not limit the allergens to be used for diagnosis to the a few representatives of protein families, is more appropriate for a personilized diagnosis than CRD.

## Structure–function relationships in allergenic proteins

The immunological processes leading to the development of the allergic disease are strongly associated with the structural features of each allergen. In the last few decades, the exploitation of molecular biology, crystallographic studies and molecular modeling has provided a significant amount of knowledge concerning the structure of several allergens, and of their regions and the amino acid residues involved in immunological functions and interactions with other molecules of the immune system [38–44]. For instance, the elucidation of the crystal structure of kirola [45], the kiwifruit allergen Act d 11, has allowed us to understand the reason for the IgE co-recognition between this allergen and others belonging to the PR-10 family, including Bet v 1 [46]. Act d 11 was not expected to show any co-recognition with the Bet v 1-like allergens because of the sequence identity, which is generally lower than 21%. In fact, on the basis of the amino acid sequence, Act d 11 belongs to the major latex protein/ripening related protein (MLP/RRP) family. Nevertheless, the analysis of the 3D-structure clearly showed that Act d 11 has a fold very similar to that of Bet v 1 and other PR-10 related allergens regardless of the low sequence identity. Then, the observation that in the IgE-binding regions the number of residues shared by Act d 11 and Bet v 1-like proteins is generally more than 35%, and sometimes more than 50%, suggests a conservation of epitope regions and may explain the detected IgE co-recognitions.

The structural basis of immunological cross-reactivity and the structural determinants underlying the strong sensitization potential of major allergens have also been investigated by analysis of the structural and functional properties of several allergens. For instance, a comparative functional study of Pru p 3 and Cor a 8, using 12-mer peptides covering the sequence of the two proteins, revealed that the peach LTP initiates the sensitization process and that the T cell reactivity to the hazelnut homolog is predominantly based on cross-reactivity with Pru p 3 [47].

The lower allergenic potential of Cor a 8 was attributed to the higher sensitivity of its structure to the lysosomal proteases.

## Allergens as reagents in allergy diagnosis

Allergenic proteins represent a critical reagent in allergy diagnosis. They have all the chemical and physical properties inherent in protein molecules and therefore are dynamic objects the structure of which is affected and modelled by different environmental/experimental conditions, including temperature, salts, pH, medium polarity and interacting molecules. In addition, some recombinant allergens do not have the same structure when compared to their natural counterparts because they sometimes have incorrect disulfide bridges or lack post-translational modifications. Allergy diagnostic tests try to reproduce, and display, what happens in vivo when the specific IgE produced by allergic patients recognizes and binds specific regions of an allergenic protein, namely the antigenic epitopes of the allergen. Nevertheless, the experimental conditions of the diagnostic tests cannot reproduce exactly those occurring in living organisms. The knwoledge of these mechanisms can help the interpretation of discrepancies observed when results obtained using different diagnostic methods, or different preparations of the same allergen, are compared.

Allergens are recognized by specific IgE when the target epitopes are exposed on the molecule surface and the binding is chemically and physically allowed. Any condition that can alter the protein conformation, the charge of chemical groups and the access to epitopes can have an effect on the IgE binding. This is valid for both linear sequence and conformational epitopes, but different proteins appear differently sensitive to environmental factors. Some proteins, like the transcription factor RfaH from *Escherichia coli* [48, 49], are able to undergo extreme conformational changes under certain conditions, moving to the interior the amino acids that were on the surface. The intrinsically disordered proteins (IDP), including the allergen Man e 5 from manioc [50], and hybrid proteins, like mammalian serum albumin [51], containing ordered and intrinsically disordered regions (IDPRs), provide additional examples of protein three-dimensional structure plasticity.

Examples of allergens displaying structural changes as a function of environmental/experimental conditions can be found in the literature. For instance, Phl p 7, the polcacin, a calcium-binding protein from timothy grass pollen, showed three different conformations when analyzed in solution by NMR in different environmental conditions [52] Kiwellin, Act d 5, is a kiwifruit allergenic protein displaying variable structural and immunological features. Structural studies highlighted a strong dependence of the Act d 5 properties on environmental conditions. For instance, this protein is more structured in low polarity media and at low pH [53, 54]. The testing of a population of twenty-nine subjects allergic to kiwifruit using two different ST protocols, a standard one where Act d 5 was maintained at neutral pH and an alternative one with the allergen exposed at the acidic pH of the natural source, produced different results. In fact, three patients (10%) had a reaction only to Act d 5 at acidic pH, three (10%) only to the allergen dissolved in a neutral solution, and five (17%) showed a reaction to Act d 5 solubilized in both conditions. In addition, all the analyzed twenty-nine subjects were negative when tested on Act d 5 immobilized on the ISAC biochip [32] These results were explained by assuming that the assay conditions influenced the results of the diagnostic systems by affecting the protein structure and modulating the pattern of exposed antigenic epitopes.

Different patterns of IgE-binding epitopes can also be detected using different methods, like IgE dot blotting and immunoblotting. For instance, the results obtained with Pun g 1 and pommaclein (Pun g 7) in dot blotting were not always correlated with those in immunoblotting [5]. It is conceivable that these discrepancies were due to the different profiles of conformational and linear sequence epitopes available for IgE binding in the two experimental conditions.

Overall, these observations suggest that in allergy diagnosis the use of a combination of different conditions and procedures can increase the number of epitopes available for specific IgE detection.

## What clinicians should know

### One size does not fit all

Precision Medicine Initiative^®^ (PMI) [55] is a project targeted to the needs of individual patients. PMI applied to allergology includes the stratification of patients, based on their diversity in age, sex, race/ethnicity and geographic/socioeconomic status, all features that might differentiate a given patient from others with a similar clinical disease. Moreover, while analyzing patients with diseases allows a systematic study of the disease outcomes, the study of sensitized but healthy patients can contribute to identifying new risk factors predictive of future allergic reactions [55, 56].

**Fig. 3:**
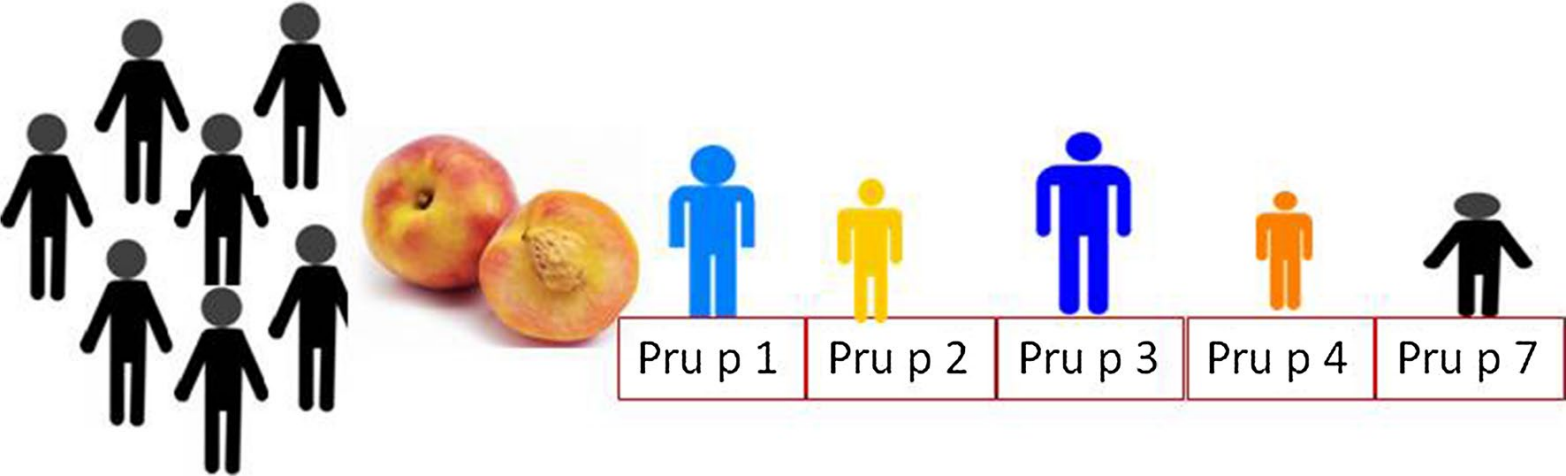
Peach allergic patients: “one size does not fit all”

With PMI the allergists have to deal with molecular mechanisms of allergy and they have to characterize the endotype of any single patient [57, 58] renouncing the idea that a suspected allergic patient should be tested either towards “the most common allergenic sources” only or towards targeted specific IgE based on the patient’s history [59, 60]. What can be statistically attributed to <1% of a population can become 100% critical for a single specific patient. This implies that the allergy investigation should be carried out in the most extended and comprehensive way possible [3, 35]. For example in the case of peach allergy, considering as similar all patients showing specific IgE to peach could be very erroneous. So far five allergenic proteins have been described in the peach fruit: Pru p 1 (Bet v 1 like), Pru p 2 (thaumatin-like protein), Pru p 3 (9 k-LTP), Pru p 4 (profilin), Pru p 7 (gibberellin-regulated protein) (www.allergome.org) (Fig. 3). Pru p 1 was found to be responsible for the oral allergy syndrome characterized by lip angioedema, oral cavity itching, tightness of the throat and itching in the ear in approximately 70% of patients allergic to Betulacee/Fagales pollen [61, 62].

Pru p 2 can be recognized by human IgE, as happens for all thaumatin-like proteins, but further studies are required to evaluate its precise role in food allergic reactions since mono sensitized/allergic patients have not yet been described [63]. Pru p 3 is the LTP sensitizer showing the highest prevalence responsible for generalized reactions [37] LTPs are pollen and food allergens widely distributed in plants. These proteins constitute a family of molecules with a molecular mass of 7 kDa (7 k-LTP subfamily) or 9 kDa (9 k-LTP subfamily) [20]. They share variable levels of primary and three-dimensional structure similarities underlying cross-reactions among the components of the family. Later studies underlined that LTP is ubiquitous in the plant. Based on this observation, during our clinical activity, we have recorded how many patients have eliminated all foods containing an LTP from their diet because of the Pru p 3 sensitization without further testing for other LTPs. This has led to a decrease in the quality of the patient’s life and an unjustified fear of eating. Indeed, the real life clinical experience shows that generally people allergic to Pru p 3 can eat most plant foods, with only a few exceptions. This is the best demonstration that cross-reactions between LTPs frequently occur, but the clinical reactivity can be very different. In addition, it has become clear that Pru p 3 cannot be used in diagnosis as “the marker” to detect allergy to all the proteins of the LTP family. In line with the patients’ reports, molecular tests reveal individual patterns of sensitization to LTP from different sources [32]. Frequently Pru p 3 is included in the group of LTPs recognized by individual allergic subjects, although sometimes the peach LTP is excluded. Mono sensitizations to individual analyzed LTPs have also been detected. These observations highlight that the best results (in terms of safety and avoidance of unnecessary deprivations) can be obtained by using the highest possible number of LTPs in diagnostic tests [64]. Severe generalized allergic reactions can also be due to Pru p 7 alone or in association with Pru p 3 [27, 65] whereas Pru p 4 can cause the oral allergy syndrome [66]. Peach extracts (peel and/or pulp) also contain cross-reactive carbohydrate determinants (CCD). The in vitro specific IgE assays on extracts can provide positive results caused by those CCD, but without any real allergy to peach [67, 68]. In this case, the presence or the absence of sensitization to known peach protein specific IgE is fundamental to the interpretation of such in vitro reactivity.

## Unmet needs

It is well known that allergen extracts are limited in terms of potency, heterogeneity, reproducibility and quality control; they are unable to differentiate genuine allergens from cross reactive allergens, particularly in polysensitized patients with broad IgE antibody repertoires. At the same time many single allergenic proteins contained in each allergenic source are not yet identified and characterized, and therefore, not currently available for allergy diagnosis. In addition, there are cases in which the clinical significance of some protein-IgE recognitions is not clear. This has led some authors to claim that the microarray panels of molecular allergens currently available detect unwanted or unneeded IgE antibody specificities, thus providing difficult challenges in the interpretation of the results [69, 70]. We would suggest putting the question in the opposite way: is there any IgE antibody specificity which really merits being ignored? As a paradigmatic example in another medical field, is finding hyperglycemia in an asymptomatic patient unnecessary information or could it be useful for prevention? Do we really want to perform an early diagnosis in not yet affected patients or do we just want to diagnose the diseases we already shown? A comprehensive diagnostic approach implies first detecting all the patient’s sensitizations and then, based on clinical history and food challenges if needed, establishing the foods to be avoided. Specific IgE, even in the absence of allergy, could be a risk factor for future clinical reactions, or the memory of a previous allergic status [70].

## Research and solutions

Ideally, a diagnostic system for allergy should contain all the allergenic proteins to which a subject might be exposed and each molecule should bear on the surface all the IgE binding epitopes. In reality, this is not possible now and probably never will be. In fact, we would have to have a huge number of isolated proteins available for testing in a single system, and each molecule in the functional conformation. Nevertheless, diagnostic systems increasingly similar to the ideal one can be realistically designed and produced.

The FABER test [71] is a new multiplex nanotechnology–based diagnostic system for allergy diagnosis with the highest number of allergens currently available. This is a flexible system that can be easily extended in terms of the number of allergens when additional ones are available for diagnosis. The first version of this multiplex diagnostic system, FABER 244, contains 122 purified and characterized allergenic proteins (e.g. panallergens and genuines) and 122 protein extracts (e.g. from pollens, mites, epithelia, mould and animal- and plant-derived foods). FABER represents the first test which combines the advantages of “tradition” (protein extracts) with those of “modernity” (isolated molecules). Molecular allergology clearly offers many advantages when compared to the use of protein extracts. However, the number of isolated allergens available for diagnosis is still too limited [72] Therefore, at least at this stage, the use of extracts can be considered a substitute for allergenic molecules not yet identified or not yet available for diagnosis.

Unlike the first generation of microarray-based tests, FABER allows the customized immobilization of each allergen to specific nanobeads. Allergen-conjugated nanobeads are then arrayed on a solid surface ready for the subsequent testing phase. Based on the biochemical features of the allergens, the conjugation with the nanobeads is carried out using optimized protocols. Furthermore, the function of bound allergens is controlled and, when necessary, a combination of different immobilization conditions are used, in order to increase the number of epitopes available on the molecule surface and useful to improve the performance of the specific IgE detection. A major issue in high throughput testing systems is the production of many results. Addressing this problem in real life clinical activities and with the aim of helping patients to understand better their allergies, a new electronic tool has been developed: the CAAM Digital Reporting System (CDRS) [71]. CDRS is a tool accessible from personal computers, tablets and smart phones, giving an online dynamic visualization of the individual’s own allergy test result, allowing patients and doctors to deal with the hundreds of results and related information simply by touching the screen. Recently, a professional version of the same tool, the CDRS PRO, has been released which offers information, tutorials, and examples of rare allergy cases to specialists.

The combination of nanotech plus allergenic molecules plus information and communication technology which has been used to create the FABER test will lead to an increase in the comprehensiveness of testing almost without limitations.

## Conclusion

Bearing carefully in mind that the presence of specific IgE (sensitization) does not necessarily predict clinical allergy, it is anyway well accepted that specific IgE are detectable in the blood even years before symptoms become clinically evident. The history of medicine proves that screening for common and unusual diseases is the winning approach leading to the earliest opportunity to take action on a disease.

Molecular Allergology is providing a fundamental support to the ongoing improvements of allergy diagnosis. The knowledge of allergen identity and structural features can find several practical applications in the field of diagnosis, immunotherapy and overall patient management. About two decades of research and application of Molecular Allergology has definitely led to great changes in this field. Precision medicine in the field of allergy is based on the concept of diagnosis at the molecular level leading to personalized treatments. What we have learned suggests that the number of allergenic molecules not yet identified and/or characterized is greater than those currently known. X-ray studies and functional tests on allergen fragments have provided valuable insights about the IgE-binding structural determinants. However, the elucidation of the crystal structures does not provide information on the conformational changes occurring in solution and in different environmental conditions that can strongly affect the epitope set exposed on the allergen surface and available for IgE binding. The valuable data provided by the elucidation of the crystal structures of allergens should in the future be enriched with studies in solution by NMR [73]. In fact, the use of this methodology, rather than crystallography, allows the investigation of the IgE-allergen interaction in conditions that are more similar to those encountered in vivo. In prospect, we expect that in the future new allergens will be identified and characterized including studies in solution in conditions as similar as possible to the physiological ones. This new knowledge will be able to contribute to a progressive improvement of test systems, allergy diagnosis and patient treatments.

### The three take home messages

- The knowledge of the structural features, including IgE-binding determinants, of allergenic molecules provides important insights for allergy diagnosis and immunotherapy.
- “One size does not fit all”. The diagnosis of each allergic disease has to be personalized in order to be optimized, to tailor the preventive measures and to reduce the cost for the patients and society.
- The history of medicine proves that screening for common and unusual diseases is the winning approach leading to the earliest opportunity to take the most appropriated actions on a disease, bearing carefully in mind that the presence of specific IgE (sensitization) does not necessarily imply Allergy.

## Abbreviations

CAAM: Centri Associati Allergologia Molecolare
CDRS: CAAM Digital Reporting System
LTP: Lipid transfer protein
NMR: Nuclear magnetic resonance
PMI: Precision Medicine Initiative
ST: Skin test
